# Supplementary material for: SNP genotypes in CYP2C9 and VKORC1 genes do not affect prostate cancer or cancer mortality among warfarin users in Finnish prostate cancer patients
Source: PLoS One. 2025 Oct 7;20(10):e0329438. doi: 10.1371/journal.pone.0329438 (PMC12503347; doi:10.1371/journal.pone.0329438)
Supplement: Attachment — Submitted filename: RESPONSES TO REVIEWERS PLOS ONE.docx [file pone.0329438.s005.docx]

**CORRESPONDING FOR MANUSCRIPT EVALUATION**

We are thankful for all suggested improvements made to our current study. We have now gone through all the comments and requirements, responses the questions raised and update the manuscript accordingly.

SPECIFIC REQUEST FROM EDITORIAL OFFICE

In particular:

- language problems should be removed

Response: We have now solved language problems raised by reviewer 2.

- presentation problems must be fixed

Response: We have now solved presentation problems raised by the editorial office and reviewers.

- details of the cohort description should be made clear

Response: We have now clarified our cohort description clearer and added information on data quality assessment.

- information about the statistical post-hoc analysis and about data distributions would increase the value of the manuscript

Response: We have added information of the statistical test of interaction term calculation used (Wald test). Also, we conducted statistical analysis comparing population characteristics of different SNP grouping.

- internet links used in the references list should be verified and updated ([1] - link is not actual; are Authors sure that such unstable and not reviewed sources should be used as references?).

Response: The link used here leads to the updated webpage including statistics of world cancer observation collected by World Health Organization (WHO). We have changed the link for relevant publication. We also noted that reference [29] was a link to the database which is open only with credentials, thus, we removed the reference from the list.

RESPONSES TO EDITORIAL OFFICE REQUIREMENTS:

Journal Requirements:

Requirement 1. Please ensure that your manuscript meets PLOS ONE's style requirements, including those for file naming. The PLOS ONE style templates can be found at

https://journals.plos.org/plosone/s/file?id=wjVg/PLOSOne_formatting_sample_main_body.pdf and

https://journals.plos.org/plosone/s/file?id=ba62/PLOSOne_formatting_sample_title_authors_affiliations.pdf.

Response: Thank you for providing the links for formatting samples. We have now updated our manuscript according to these instructions.

Requirement 2. Thank you for stating in your Funding Statement:

“FinRSPC has been supported by grants from Academy of Finland (grant #260931), Cancer Foundation Finland sr, and Expert Responsibility Area of the Pirkanmaa Hospital District (grant #9V065) (A.A). This study was supported by grant from Cancer Foundation Finland (grant #220055) (T.M). The funders had no role in the study design, data collection and analysis, decision to publish, or preparation of the manuscript.”

Please provide an amended statement that declares *all* the funding or sources of support (whether external or internal to your organization) received during this study, as detailed online in our guide for authors at http://journals.plos.org/plosone/s/submit-now. Please also include the statement “There was no additional external funding received for this study.” in your updated Funding Statement.

Please include your amended Funding Statement within your cover letter. We will change the online submission form on your behalf.

Response: We have now updated the Funding Statement, removed it from manuscript file and added the updated version to a part of our cover letter of revised manuscript.

Requirement 3. We note that you have indicated that there are restrictions to data sharing for this study. For studies involving human research participant data or other sensitive data, we encourage authors to share de-identified or anonymized data. However, when data cannot be publicly shared for ethical reasons, we allow authors to make their data sets available upon request. For information on unacceptable data access restrictions, please see http://journals.plos.org/plosone/s/data-availability#loc-unacceptable-data-access-restrictions.

Before we proceed with your manuscript, please address the following prompts:

a) If there are ethical or legal restrictions on sharing a de-identified data set, please explain them in detail (e.g., data contain potentially identifying or sensitive patient information, data are owned by a third-party organization, etc.) and who has imposed them (e.g., a Research Ethics Committee or Institutional Review Board, etc.). Please also provide contact information for a data access committee, ethics committee, or other institutional body to which data requests may be sent.

b) If there are no restrictions, please upload the minimal anonymized data set necessary to replicate your study findings to a stable, public repository and provide us with the relevant URLs, DOIs, or accession numbers. Please see http://www.bmj.com/content/340/bmj.c181.long for guidelines on how to de-identify and prepare clinical data for publication. For a list of recommended repositories, please see https://journals.plos.org/plosone/s/recommended-repositories. You also have the option of uploading the data as Supporting Information files, but we would recommend depositing data directly to a data repository if possible.

Please update your Data Availability statement in the submission form accordingly.

Response: We have now updated our Data Availability statement in the manuscript accordingly: There are legal restriction to share the data openly as our data is considered sensitive personal health data. Data access can be applied from Finnish Social and Health Data Authority, Findata (contact information: <https://findata.fi/en/> and info@findata.fi).

Requirement 4. In the online submission form, you indicated that your data is available only on request from a third party. Please note that your Data Availability Statement is currently missing [the name of the third-party contact or institution / contact details for the third party, such as an email address or a link to where data requests can be made]. Please update your statement with the missing information.

Response: We have now added this information to our data availability statement (see above).

5. Thank you for stating the following in the Acknowledgments Section of your manuscript:

“FinRSPC has been supported by grants from Academy of Finland (grant #260931), Cancer Foundation Finland sr, and Expert Responsibility Area of the Pirkanmaa Hospital District (grant #9V065) (A.A). This study was supported by grant from Cancer Foundation Finland (grant #220055) (T.M). The funders had no role in the study design, data collection and analysis, decision to publish, or preparation of the manuscript.”

We note that you have provided additional information within the Acknowledgements Section that is not currently declared in your Funding Statement. Please note that funding information should not appear in the Acknowledgments section or other areas of your manuscript. We will only publish funding information present in the Funding Statement section of the online submission form.

Please remove any funding-related text from the manuscript and let us know how you would like to update your Funding Statement. Currently, your Funding Statement reads as follows:

“FinRSPC has been supported by grants from Academy of Finland (grant #260931), Cancer Foundation Finland sr, and Expert Responsibility Area of the Pirkanmaa Hospital District (grant #9V065) (A.A). This study was supported by grant from Cancer Foundation Finland (grant #220055) (T.M). The funders had no role in the study design, data collection and analysis, decision to publish, or preparation of the manuscript.”

Please include your amended statements within your cover letter; we will change the online submission form on your behalf.

Response: We have now removed Funding Statement from the manuscript file and added updated statement as a part of our cover letter.

Requirement 6. Please review your reference list to ensure that it is complete and correct. If you have cited papers that have been retracted, please include the rationale for doing so in the manuscript text, or remove these references and replace them with relevant current references. Any changes to the reference list should be mentioned in the rebuttal letter that accompanies your revised manuscript. If you need to cite a retracted article, indicate the article’s retracted status in the References list and also include a citation and full reference for the retraction notice.

Response: We are thankful for your suggestions and have now gone through our references. Unfortunately, we did not find any retracted publication from the reference list (based on screening using PubMed). If there indeed is one, we can replace it with another one, however, editorial office is asked to point out which reference is under this concern. We found one publication with erratum and information of this is now added to the reference list [24].

RESPONSES TO REVIWERS COMMENTS:

Reviewer #1: This text describes the population characteristics of CYP2C9 and VKORC1 genotypes in two study cohorts. The content is clear and logically sound, but there are still some shortcomings and suggestions for improvement:

Comment: There is a lack of logical connection between the discussion of prostate cancer risk factors and the coagulation cascade between lines 52 and 53 on page 3. The sections discussing the in vitro studies of warfarin and the current epidemiological situation between lines 60-65 need to be refined for clarity.

Response: Thank you for these comments.

We have expanded our introduction on how coagulation cascade may aid cancer cells and modified the text for greater clarity. Introduction, 2^nd^ and 3^rd^ paragraph:

“The coagulation cascade has been proposed to play a role in hematogenous spread of cancer and formation of metastases. Platelets adhere to circulating cancer cells, protecting them from the immune system [5-7]. Platelet RNA is altered in cancer patients, and platelets could serve as potential cancer biomarker [8]. Therefore, it would be logical to assume that anticoagulants, which affect platelet function, would decrease metastatic spread by reducing platelet protection of circulating cancer cells.

Previous pre-clinical studies have reported that widely used anticoagulant warfarin may protect against tumor cell spreading by mechanism related on modulation of anticoagulation cascade rather than a direct impact on cancer cells [9-11]. HFor PCa, warfarin use has been associated with decreased risk in one epidemiological study [12], but not all studies agree [13]. Furthermore in epidemiological studies of cancer mortality, the risk of cancer death is increased among warfarin users compared to non-users [14-16]. “

Comment: Although the methods section mentions that data is sourced from the national cancer registry and hospital records, there is a lack of discussion regarding data quality assessment when describing how to "supplement" cancer diagnosis information. The specific values of the International Normalized Ratio (INR) are not provided, which prevents the classification of patients into good responders and poor responders for analysis. INR is an important indicator for evaluating the anticoagulant effect of warfarin, and the absence of this data may affect the in-depth analysis of warfarin dosage adjustment and efficacy assessment.

Response: We have now added information of the data quality of Finnish register data to the material and method section (page 5, lines 98-100 and 105-108). We agree that INR values are important indicator for evaluation of anticoagulation efficacy of warfarin. Unfortunately, we had no access to the INR values, and could not use them to analyse response to warfarin therapy. We mention this point to our Discussion on study limitations, page 20, lines 14-15:

“However in these data sets, we did not have access on INR values, thus, these analyses need to be done using other data sets.”

Comment: The first part of the results mentions differences between genotypes and clinical standards (such as Gleason scores, tumor stages, etc.), but it does not discuss the clinical significance or potential reasons for these results. Please supplement with relevant research evidence.

Response: We have now added information why cases are more severe in the Hospital cohort than in the Screening cohort (page 10, lines 201-207):

“Differences in the clinical parameters of PCa between the cohorts can largely be attributed to the nature of the cohorts themselves. The Hospital cohort comprises patients with symptom-detected PCa,. Consequently, this cohort includes higher proportion of aggressive and advanced cases, with fewer low-grade PCa cases. In contrast, the Screening cohort consists of PCa cases identified through population-level PSA screening program. Most screening-detected PCa cases were asymptomatic and involved low-grade tumors.”

Comment: The results list the hazard ratios (HR) and confidence intervals (CI) for different genotypes, but the clinical significance and biological mechanisms of these results are not discussed, especially regarding why certain genotypes are associated with mortality risk while others are not.

Response:

All tested SNP genotypes affect warfarin metabolism. Observed differences between them are hypothesis-generating prompting further study to elucidate the mechanistic differences. Currently there is too little evidence to discuss why them.

Instead, we discuss generally why warfarin metabolism might affect prostate cancer outcomes and what it would mean clinically.

Reviewer #2:

Comment:

==Language problems==

1. pp 3-4, lines 67-68: Missing article, recommended change: ‘Warfarin targets Vitamin K epOxide Reductase Complex subunit 1 (VKORC1) by inhibiting its activity, an enzyme involved in the vitamin K cycle.’

2. p 5, line 109: Recommended change for improving the readability: ‘The data were accessed for research purposes between January 1, 2022, and October 31, 2024.’

3. p 7, line 140: ‘Only 0.9% of subjects were carrier’ => ‘Only 0.9% of subjects were carriers’

4. Caption of Figure 2: ‘warfaring-metabolizing’ => ‘warfarin-metabolizing’;

5. Caption of Table 4: ‘Data is expressed as hazard ration’ => ‘Data is expressed as hazard ratios’;

Response: We are thankful for careful proofreading of our manuscript. All above mention language problems have been corrected as requested.

==Presentation problems==

Comment: 1. p 9, lines 188-193: Table 1: Lacking indication that the numbers in parentheses represent the percentages for the row ‘n of men’. Some numbers in the table don’t sum up to the values declared in the caption, e. g. n of men in the screening cohort. Moreover, it is unclear what was intended to be shown regarding the data stratification by SNP type. The description in the caption suggests that the stratification is held in the table, whereas the table itself presents the summed data. It’s recommended to harmonize the description and the tabular data in this context.

Response: Thank you for these comments. We have now added missing information to the table and clarified how stratification was done. We have also clarified information of the values in the caption and now numbers are the same in the caption and in the table.

Comment: 2. Figure 1: It is recommended to improve the readability of the cause of death mapping on the graph either by enlarging the font of the geometric figures or by adding color as an auxiliary mapping.

Response: Thank you for this notion. We have revised the Figure to improve readability

Comment: 3. Figure 2: The recommendations are similar to those for Figure 1. Apart from that, the figure does not clearly reflect the stratification declared in the caption. It can be deduced from the caption that the second, third, and fourth graphs, as viewed from top to bottom, pertain to warfarin users referenced to the non-users but this information may be overlooked by the reader when scanning the article. Thus, it might be helpful to include this reference in small font individually below the captions of each graph. Additionally, what about the division into two cohorts? Consistently with the caption of Figure 1, I would expect the separate presentation of the results for each of these cohorts. Are they summed up in the Figure 2? If so, it is recommended to indicate this in the caption.

Response: We have now added reference group and title for the whole figure to the Fig 2. Hopefully this clarifies the message of the image. Only Screening cohort was used to evaluate how warfarin user status impact on the hazard ratios. We have now clarified that in the title and caption.

Comment: 4. Table 2: Adding information to the caption that ‘Ref’ stands for ‘reference group’ would improve the readability of the table. Similarly to the Table 1, it is also recommended to check whether the summations of cases are correct within the cohorts and genotypes.

Response: We have now added information of reference group into the titles. We have gone through all our summations of the cases and corrected the information to the text and tables. Altogether, we have 810 and 1527 cases in each cohort, however, information of CYP2C9 SNP distribution was available only for 790 and 1437 cases, thus, analyses where these SNPs were evaluated was limited to lower number of PCa cases. This explains the differences in the summed values in the tables. We have now clarified this in the text (page 7, lines 146-150) and titles of the tables.

==Content-related questions and recommendations==

Comment: 1. p 6, lines 113-114: ‘The study cohort was linked to the national prescription database of the Social Insurance Institution of Finland …’. It is recommended to make this description more precise regarding the cohort or cohorts it refers to. Does this description refer to the screening cohort, the hospital cohort or both? Another fragment which occurs in the lines 143-146 suggests that only screening cohort is concerned but it would be with benefit to readability to mark this information explicitly.

Response: Thank you for this comment. We have now clarified that only Screening cohort was linked to the prescription database (page 6, line 121).

Comment: 2. p 8, lines 164-165: ‘Interaction was statistically tested including interaction term between warfarin use and SNP genotype into Cox regression model’. Please provide the name of the test used for the evaluation of the interaction term: is it Wald test, Likelihood Ratio Test or other?

Response: We have now added this information to the Statistical analyses section in Material and Methods (page 8, line 154). We used Wald test.

Comment: 3. p 8, lines 177-178: ‘otherwise the distributions of wild type and heterozygous SNP carriers were similar in the two study cohorts’. These frequencies are indeed similar at a glance, but it would be advisable to test the independence of the aforementioned distributions by e.g. Chi-squared test for greater precision and provide the calculated p-value. This remark is more general and refers to other fragments where distributions/frequencies are compared.

Response: We have now tested distribution between grouping in the Table 1 using Chi-Square test for categorical variables and ANOVA for linear variables. Based on this statistical testing, there were no differences between the groups. We have now added this information to manuscript section ‘Statistical analyses’ (page 9, lines 184-185) and to the caption of the table.
