# Supplementary material for: SNP genotypes in CYP2C9 and VKORC1 genes do not affect prostate cancer or cancer mortality among warfarin users in Finnish prostate cancer patients
Source: PLoS One. 2025 Oct 7;20(10):e0329438. doi: 10.1371/journal.pone.0329438 (PMC12503347; doi:10.1371/journal.pone.0329438)
Supplement: Attachment — Submitted filename: Responses to reviewers comments.docx [file pone.0329438.s007.docx]

Responses to reviewers’ comments

July 9^th^, 2025

We are thankful for the comments and made modifications to our manuscript based on them. All changes are marked at the revised version of the manuscript. Please, below you can find our responses to reviewers’ comments.

Review Comments to the Author:

Comment: Reviewer #2: The authors have addressed the recommendations outlined in my review and implemented the suggested improvements. Therefore, I recommend the acceptance of the submitted paper.

Response: Thank you for your positive feedback.

Comment: Reviewer #3: All comments have been addressed and I have no further comments to discuss with authors or editors on this manuscript.

Response: Thank you for your positive feedback.

Reviewer #4:

Comment: 1. There are some grammatical, alignments and typographical errors are noted in the manuscript and it should be thoroughly checked and corrected throughout the manuscript.

Response: We have now checked and corrected the text from grammatical, alignments and typographical errors.

Comment: 2. Check the abbreviations throughout the manuscript and introduce the abbreviation when the full word appears the first time in the abstract and the remaining for the text and then use only the abbreviation (For example, Prostate cancer (PCa), CVD, etc.,). Make a word abbreviated in the article that is repeated at least three times in the text, not all words to be abbreviated.

Response: We have now gone through the manuscript and clarify the use of abbreviations throughout the manuscript.

Comment: 3. In introduction, the authors may cite prevalence or incidence data “Prostate cancer” and it should be in either 2024 or 2025.

Response: Unfortunately, the latest statistics of prevalence and incidence rates are from the year 2022 (Bray F et al. Global cancer statistics 2022: GLOBOCAN estimates of incidence and mortality worldwide for 36 cancers in 185 countries. CA Cancer J Clin. 2024 May-Jun;74(3):229-263.). We have cited the publication using data from 2022 which estimate the current numbers. Related on prostate cancer incidence data, in our opinion, the reference used represents the current situation.

Comment: 4. When referring to SPSS versions beginning from 19, authors should cite ‘IBM SPSS Statistics for Windows, version 21 (IBM Corp., Armonk, N.Y., USA)'.

Response: As suggested, we have now added more detailed information on the statistical programs we used (page 9, lines:189-190).

Comment: 5. The version of the software used for statistical investigation (Stata) in the present investigation should be given under the heading “Statistical analysis”.

Response: We have added the requested information under the heading of Statistical analysis (page 9, line:190).

Comment: 6. The authors may improve the discussion of their results by focusing on the present findings and introducing data from other authors who also worked with the same or other studies with recent references since it is lack of sufficient references.

Response: We have now added more comprehensive discussion on reflection of our findings on previous knowledge of the topic (page: 17, lines:11-20):

“To our knowledge, this is the first study to evaluate the association of SNPs rs1057910, rs1799853, and rs9923231 with PCa progression. However, SNP rs1057910 has previously been associated with an increased risk of developing colorectal carcinoma [33]. We did not assess whether the investigated SNPs are associated with the risk of developing PCa, therefore, this should be explored using other cohorts that include genotyping data from healthy controls as well. In PCa, VKORC1 gene expression has been studied in prostate tissue, and it was found to be higher in benign tissue compared to PCa tissue. Interestingly, SNP rs2359612, which also influences warfarin dosing, was linked to PCa risk [34]. Notably, based on a genome-wide association study (GWAS), none of the investigated SNPs are among the 451 independent variants reported to be associated with PCa risk [35].”

Reviewer #5: The manuscript titled "SNP genotypes in CYP2C9 and VKORC1 genes do not affect prostate cancer or cancer mortality among warfarin users in Finnish prostate cancer patients" is well-conducted. I read the previous comments from the reviewers. I have some additional comments for improvement:

Comment: 1- Define abbreviations in their first use and make sure that abbreviated forms are used after the definition.

Response: We have now gone through the manuscript and clarify the use of abbreviations throughout the manuscript.

Comment: 2- In the discussion, the limitations and strengths should be in a separate heading, just before the conclusions.

Response: We have added separate headings as suggested (page 19).

Comment: 3- The discussion section is not comprehensive. Add more data from previous meta-analysis and compare them with your findings.

Response: We have now added more comprehensive discussion on reflection of our findings on previous knowledge on the topic. We have also compared our findings to a recent GWAS meta-analysis of gene variants associated with prostate cancer risk (page: 17, lines:11-20).

“To our knowledge, this is the first study to evaluate the association of SNPs rs1057910, rs1799853, and rs9923231 with PCa progression. However, SNP rs1057910 has previously been associated with an increased risk of developing colorectal carcinoma [33]. We did not assess whether the investigated SNPs are associated with the risk of developing PCa, therefore, this should be explored using other cohorts that include genotyping data from healthy controls as well. In PCa, VKORC1 gene expression has been studied in prostate tissue, and it was found to be higher in benign tissue compared to PCa tissue. Interestingly, SNP rs2359612, which also influences warfarin dosing, was linked to PCa risk [34]. Notably, based on a genome-wide association study (GWAS), none of the investigated SNPs are among the 451 independent variants reported to be associated with PCa risk [35].”

Comment: 4- Mention the clinical utility of your findings in the discussion.

Response: Thank you for pointing this out. We have now added the sentence clarifying this point in the beginning of the discussion (page: 17, lines: 10-12).

“Thus, this indicates that the genetic factors influencing warfarin dosing do not impact the potential benefits cancer patients may receive from using warfarin.”

Comment: 5- I found some typos and grammatical errors. A native review is warranted.

Response: We have now checked and corrected the text from grammatical, alignments and typographical errors.

Comment: Reviewer #6: I think the paper is sound and all comments have been addressed. I think in general the paper is solid and should be published. Besides the results are very usefull for the scientific community involved in this subject.

Response: Thank you for your positive feedback.
